# LVN-led Transitional Care Coordinator Program for Hip Fractures Reduces Mortality

**DOI:** 10.1093/geroni/igaf122.3343

**Published:** 2025-12-31

**Authors:** Peter Aldo Giammanco, Veranika Mejbaryan, Kathleen Breda, Michelle Keller, Carol Lin

**Affiliations:** California University of Science and Medicine, Colton, California, United States; Cedars-Sinai, Los Angeles, California, United States; Cedars-Sinai Medical Center, Los Angeles, California, United States; University of Southern California, Los Angeles, California, United States; Cedars-Sinai Medical Center, Los Angeles, California, United States

## Abstract

Hip fractures pose increased risk of morbidity and mortality in geriatric patients due to complex postoperative. This study analyzed the effects an LVN-led transitional care program had on outcomes of geriatric hip fracture patients in a large urban health system. A cross-sectional study with a non-randomized control group was performed from July 2018 to April 2024. An LVN was trained to be a transitional care coordinator (TCC) for the academic orthopaedic faculty, provided with postoperative protocols for pain, mobilization, DVT prophylaxis, and wound care following hip fracture surgery, and performed phone calls to patients at 7-, 14-, 30-, and 90-days post-discharge. The TCC either answered patient questions directly or escalated questions to the orthopaedist or PCP. The study and control groups included patients contacted by the TCC (TCC) and not contacted by the TCC (control). Bivariate analyses were conducted regarding 90-day ED visits, 90-day readmissions, 90-day mortality, and 1-year mortality. A total of 1569 patients underwent hip fracture care during the study period, 620 (39.5%) of which included TCC. The control and TCC group were similar in age (83.13±8.73 vs. 82.58±8.73 years, p = 0.182) and sex (67.0% vs. 71.1% female, p = 0.086). No differences were seen in 90-day ED visits between control and TCC (6.9% vs 8.7%, p = 0.175) or 90-day readmissions (23.7% vs.19.7%, p = 0.06). TCC had lower 90-day (7.8% vs. 3.5%, p = 0.001) and 1-year mortality rates (11.6% vs. 7.1%, p = 0.003). These findings highlight the benefits and importance of a TCC program as it reduced morality and potentially addressed gaps of post-discharge care.

